# Novel Synthesis Method of Micronized Ti-Zeolite Na-A and Cytotoxic Activity of Its Silver Exchanged Form

**DOI:** 10.1155/2015/428121

**Published:** 2015-02-03

**Authors:** H. F. Youssef, W. H. Hegazy, H. H. Abo-almaged, G. T. El-Bassyouni

**Affiliations:** ^1^Refractories, Ceramics and Building Materials Department, National Research Centre (NRC), Dokki, Cairo 12622, Egypt; ^2^Department of Chemistry, Faculty of Science, Suez University, Suez 43533, Egypt; ^3^Biomaterials Department, National Research Centre (NRC), Dokki, Cairo 12622, Egypt

## Abstract

The core-shell method is used as a novel synthetic process of micronized Ti-Zeolite Na-A which involves calcination at 700°C of coated Egyptian Kaolin with titanium tetrachloride in acidic medium as the first step. The produced Ti-coated metakaolinite is subjected to microwave irradiation at low temperature of 80°C for 2 h. The prepared micronized Ti-containing Zeolites-A (Ti-Z-A) is characterized by FTIR, XRF, XRD, SEM, and EDS elemental analysis. Ag-exchanged form of Ti-Z-Ag is also prepared and characterized. The Wt% of silver exchanged onto the Ti-Zeolite structure was determined by atomic absorption spectra. The *in vitro* cytotoxic activity of Ti-Z-Ag against human hepatocellular carcinoma cell line (HePG2), colon cell line carcinoma (HCT116), lung carcinoma cell line (A549), and human Caucasian breast adenocarcinoma (MCF7) is reported. The results were promising and revealed that the exchanged Ag form of micronized Ti-Zeolite-A can be used as novel antitumor drug.

## 1. Introduction

Zeolites are inorganic, crystalline, microporous, and alumino-tectosilicates with an open framework (ring structure) of SiO_4_ and AlO_4_ tetrahedra, connected via oxygen atoms at their corner points. The three-dimensional structure of Zeolites generates uniformly sized interconnected micropores and channels which form sharply defined, two- or three-dimensional channel systems of molecular dimensions in 0.3–1.4 nm range where cations, large molecules, and even cationic groups (as water, ammonia, carbonate and nitrate anions, etc.) are present [1]. Water molecules are removable (i.e., sorbed/adsorbed) where alkali cations seem to be exchanged. These channels are identified by its direction relative to the crystallographic axes. The number of either T- (Si- and Al-) or O-atoms forming the rings controlling the diffusion through the channels and the crystallographic free diameter of the channels, in Angstrom units, is based upon the atomic coordinates of the type of materials and the oxygen radius of 1.35 Å [2].

More than 46 Zeolite minerals are naturally occurring and more than 150 synthetic structures are present in the literature. Synthetic Zeolites are used commercially more than the natural ones due to their purity, degree of crystallinity, and pore-size uniformity [3]. Zeolites are usually prepared from dense gels containing silica and alumina species at elevated temperatures in a relatively expensive process. Thus, the presence of natural resources necessary for their production such as rocks, volcanic tuffs, pumice, and diatomite is a matter of economic interest.

Microwave-assisted-hydrothermal synthesis is a process that is used efficiently for the rapid synthesis of numerous ceramic oxides and porous materials [4–6]. It offers many advantages over the conventional methods, especially for Zeolite synthesis, including quick and uniform heating, homogeneous nucleation, fast dissolution of precipitated gels, and shorter crystallization time. Heating is induced via the friction of molecular motion enhanced by microwave irradiation. Thus, it is possible to heat the reactants selectively and homogeneously. Furthermore, microwave heating is energy saving and economically efficient [7, 8].

Zeolite-Na-A is one of the simplest synthetic Zeolites with a molecular ratio of 1 : 1 : 1 (Si : Al : Na). Zeolite-Na-A (ZA) exhibits the LTA (linde type A) cubic structure [9, 10]. The unit cell of Zeolite-Na-A consists of 12 SiO_2_ and 12 AlO_2_ units in a structure that conforms a large (*α*-cage) and small (*β*-cage) cavity and pore window opening with corresponding diameters of 11.4, 6.6, and 4.1 Å, respectively. The chemical composition of unit cell is Na_12_[Al_12_SiO_12_O_48_]·27H_2_O. The *α*-cage in Zeolite-Na-A accommodates 8 or 12 atoms of the total number of sodium in the unit cell, while only 4 sodium atoms are placed in the *β*-cage [9, 10]. Based on its structure and composition, Zeolite-A has high ionic conductivity and high ion-exchange capacity that facilitate its introduction in various applications such as detergency, desiccation, adsorption, separation, and ion exchange [11].

Properties exhibited by Zeolites are strongly dependent upon the exchangeable cations hosted in their framework. According to the incorporation of the metal ions into their structure, Zeolites can be either ion exchangeable or framework substituted. Introducing foreign atoms other than aluminum in the Zeolite framework structure greatly change their catalytic activity. It is well known that the aluminosilicate framework builders, T-atoms, that is, Si and Al, can be substituted by other metals such as Al^3+^ by B^3+^, Ga^3+^, and Si^4+^ or by transition metals of groups II to VII, especially Ti and V [12]. Although, it is a complicated process, the insertion of transition elements by direct synthesis into Zeolite framework is advantageous, due to the possibility of achieving a high dispersion of the metal in the Zeolitic structure. Traditionally, the introduction of cationic ions or metal clusters in Zeolites extraframework positions is performed by cationic exchange [13, 14], impregnation [15], or chemical vapor deposition [16, 17] of metal precursors after Zeolite crystallization.

Since the first direct formation of synthesized Ti-Zeolite, TS-1, in 1983 [18], with a tetrahedral titanium atoms incorporated into the framework of MFI Zeolite structure, many kinds of Ti-containing molecular sieves such as Ti-Beta have been prepared because of their interesting catalytic properties [19–23]. Both Zeolites indicated the framework Ti atoms are in tetrahedral coordination. There are two main pathways for the formation of Ti-containing Zeolites: direct hydrothermal synthesis [24–27] and postsynthesis pathway [28–32]. Direct synthesis includes the presence of Si and Ti sources in the starting gel; meanwhile, the postsynthesis method comprises a vapor phase treatment of Zeolite with TiCl_4_ known as gas-solid phase isomorphous substitution with TiCl_4_ or as CVD method [23, 29].

The synthetic difficulty of titanium incorporation into the Zeolite framework was the strong alkaline conditions for the synthesis of aluminosilicate Zeolites. Thus, most of the titanium-containing Zeolites reported so far, such as TS-1, TS-2, Ti-*β*, and Ti-MCM-41 [18, 33–35] have little aluminum in its framework. However, the synthesis of Ti-containing aluminosilicate Zeolite under strong alkaline conditions using titania-silica sol is also reported [36].

ZSM-5 was a matter of intensive investigation for the incorporation of titanium and it was found that titanium-containing Y Zeolites cannot be directly prepared by hydrothermal synthesis, but when the Zeolite [Li]Y was treated with TiCI_4_, [Li, Ti]Y was obtained with a titanium content of 12 mol% [38] which is four times higher than that in [Ti]ZSM-5. XRD, ^29^Si MAS NMR, and IR measurements indicated that dealumination of the framework occurs during tetanisation. Additional weak reflections in the XRD showed the presence of a small amount of Rutile but this does not account for all the titanium. Meanwhile, treating Al-containing Zeolite (ZSM-5) with TiCl_4_ led to the synthesis of ZSM-11 structure, which is described as TS-2 wherein some of the aluminum of the ZSM-5 is substituted by titanium [39]. In the same context, Ti incorporated Y Zeolite was prepared by postsynthesis treatment of a dealuminated USY Zeolite with an aqueous solution of (NH_4_)_2_TiF_6_ [40]. The results show that bulk Si/Al ratio was considerably larger than that of the starting Zeolite, indicating the dealumination that occurred during tetanisation process. The XRD pattern of the titanated Zeolite showed no change other than slight decrease in the peak intensities. The obtained result also presented that the Ti species were tetrahedrally incorporated into the hydroxyl nests in the Zeolite framework under acidic conditions.

The difficulty of exchanging titanium cation with native cations in Zeolites arises from the fact that titanium cation precipitates under any conditions other than extremely acidic condition (pH > 2), the same condition under which Zeolite structures are unstable and may suffer dealumination and even collapse [41]. Being the least stable Zeolite in acidic media, Ti-Zeolite Na-A is rarely reported in literature. The only recorded work was done by Kuznickl et al. [41], where a physical adsorption of TiCl_3_ is done by Zeolite-3A (K-Zeolite-A) pellets rather than by diffusion from the bulk solution.

As an inorganic material with aluminosilicate composition, Zeolites are known to possess biological activity. The incorporated water could be removed and substituted by different solutions; thus Zeolites can act as a delivery system, a process of which has already been exploited and applied in medicine [42]. Based on their unique characteristics, Zeolites are beneficial in medical and health care applications due to its biological activity, long term biological properties with chemical and biological stability [43], reversibly binding to small molecules as oxygen and nitric oxides [44, 45]. Furthermore, Zeolites well defined structures and catalytic effects make them an attractive model system for protein and enzyme mimetics [46]. They are also very effective as glucose adsorbent [47], antidiarrheal material [48], gastric antacid [49], and hypocholesterolemic [50]. Many accumulating evidences indicate their importance in regulating the immune system, as they act as nonspecific immune-stimulators similarly to superantigens [51, 52].

As antitumor active materials, natural Zeolite, particularly clinoptilolite, is proved to possess a potential role as adjuvant chemotherapy applied after initial treatment for cancer, especially to suppress secondary tumor formation [53–55]. Its micronized form is found to interfere with the lipid peroxidation in the liver of cancer-bearing mice leading to a decrease in the tumor size, improvement of the overall health status, and prolonged life span. Specifically, the finely grinded clinoptilolite inhibits protein kinase B(c-Akt), induces the expression of p21^WAFI/CIPI^ and p27^KIPI^ tumor suppressor protein, and blocks cell growth in several cancer cell lines [56].

Silver Zeolites are increasingly investigated as germicidal, bactericidal, antifungal, and antiseptic components in different compositions [57–59]. Zeolites Na-A has flavorless, odorless, and harmless properties. The ion-exchange process converts this low Si/Al Zeolites into antimicrobial candidate when replacing its Na^+^ ions with Ag^+^, Cu^2+^, and Zn^2+^ individually [60]. However, silver is the most common ion used in Zeolites-exchange process due to its stability and broad spectrum of antibacterial effects [61, 62]. The use of silver ions and metallic silver as well as silver nanoparticles can be exploited in medicine for burn treatment, dental materials, coating stainless steel materials, textile fabrics, water treatment, sunscreen lotions, and so forth. They also possess low toxicity to human cells, high thermal stability, and low volatility [63].

In our previous study [64], we found that Ag-substituted Zeolites-A has an effective antitumor effects. The* in vitro* cytotoxic results of Z-Ag against lung carcinoma cell line (A549), human hepatocellular carcinoma cell line (HePG2), colon cell line carcinoma (HCT116), and human Caucasian breast adenocarcinoma (MCF7) were reported. The results were promising and revealed that the Ag-substituted micronized Zeolites-A can be used as novel antitumor drug.

There are many reports for different biomedical applications of natural and synthetic Zeolites found in some cation forms, generally silver and zinc [53, 65–67]. No reports in the literature are recorded for antitumor activity of Ag-exchanged Ti-Zeolites Na-A.

In the present study, novel method foabr Ti-substituted Zeolites Na-A is formulated. The incorporation of Ti in the framework of Zeolites-A is achieved through a modified process of depositing thin coating layer of titanium by wet chemical method on a core of the starting Kaolin reactant in acidic conditions. Ti-coated Kaolin (Ti-K) is then thermally activated to prepare Ti-metakaolinite (Ti-MK) by calcination at high temperature before converting it into Ti-Zeolites (Ti-Z). The produced Ti-Zeolite is functionalized by silver (Ti-Z-Ag) and its cytotoxic activity is evaluated.

## 2. Materials and Methods

### 2.1. Zeolites Preparation from Kaolin

#### 2.1.1. Coating of Kaolin by Titanium Tetrachloride

The Egyptian Kaolin used in this study has the following composition: 50.54 SiO_2_, 31.48 Al_2_O_3_, 1.87 Fe_2_O_3_, and 2.26 TiO_2_ (Wt.%) and some other minor constituents containing MgO, CaO, Na_2_O, and SO_3_. Sodium hydroxide pellets (NaOH) is of analyzed ACS reagent with the composition of 98.6% NaOH + 0.4% Chloride (Sigma-Aldrich). Kaolin with particle size range between 73 and 130 nm is coated by titanium using titanium tetrachloride following the method and scheme given by Ahmed and Selim, 2011 [37]

(1) TiCl4+KaolinNH4OH→HClNH4OH  TiOH/Kaolin+NH4Cl→500–750°CTiO2/Kaolin

Based on the work of Ahmed and Selim, 2011 [37], coating of Kaolin particles by titanium proceeded by immersing certain amount of Kaolin in solutions containing different concentrations of titanium tetrachloride and hydrochloric acid, different periods of time to ensure good coverage. Ammonia solution is added dropwise to adjust the pH. The produced Kaolin paste is then filtered through a Buchner system and washed before calcination at 700°C to obtain the thermally activated, Ti-metakaolinite (TMK). The concentration of TiO_2_ in the coating layer in the starting Kaolin reaches 1.26 (Wt%) with a corresponding 0.76 (Wt%) titanium.

#### 2.1.2. Microwave Synthesis of Ti-Zeolites

Kaolin is commonly used as a starting material for synthesis of Zeolites-A since its Si/Al ratio is near to unity as that of Zeolites-A [68, 69]. Micronized Ti-Zeolites-A (Ti-Z-A), with an average grain size of less than 5 *μ*m, is synthesized from the previously prepared Ti-coated metakaolinite (TMK) using microwave irradiation at low temperature of 80°C for 2 h, following the method of Youssef et al., 2008 [70]. The concentration of NaOH used was 2 M and the solid/liquid ratio of metakaolinite to alkaline solution was 1 g/25 mL. The microwave (MARS Extraction and Digestion system, Model XP-1500, CEM Corp., Matthews, NC) is used.

#### 2.1.3. Preparation of Silver-Exchanged Zeolites

The obtained micronized Ti-containing Zeolites-A (Ti-Z-A) was modified to its Ag-exchanged form (Ti-Z-Ag) via immersing 5 g of Ti-Zeolites in 100 cm^3^ of 0.1 M solution of extra pure silver nitrate (Scharlau chemicals, Spain) at 70°C for 6 h under magnetic stirring. The solid product was well washed with 250 cm^3^ deionized water and dried at 100°C for 24 h [71].

### 2.2. Characterization Techniques

In the present study, the chemical analysis of the starting Kaolin is obtained by X-ray fluorescence using XRF instrument model AXIOS, WD-XRF Sequential Spectrometer (Panalytical, 2005). Meanwhile, the determination of the mineralogical constituents of Egyptian Kaolin, metakaolinite, and Zeolites and its Ag-exchanged form was investigated by X-ray diffraction method, using BRUKUR D_8_ ADVANE with secondary monochromatic beam Cu K*α* radiation at Kv = 40 and mA = 40. Functional groups of all materials were identified using Fourier transform infrared, FTIR, using MB154S, Bomem, Quebec, Canada equipment. Microstructures of the synthesized materials were scanned using TEM, SEM model Philips XL30 attached with EDX unit, using an accelerating voltage of 30 K.V., magnification 10x up to 400,000x, and resolution for wavelength (3.5 nm), respectively. AgNO_3_ concentration of 0.1 M exchanged onto the Zeolite was determined using atomic absorption spectra (Savant AA, GBC, Australia).

### 2.3. Cytotoxic Activity of Ag-Exchanged Ti-Zeolites

#### 2.3.1. Method

The synthesized Ti-Zeolites are supplied to the Bioassay-Cell Culture Laboratory, National Research Centre, Cairo, Egypt, for* in vitro* antitumor screening on human hepatocellular carcinoma cell line (HePG2), colon cell line carcinoma (HCT116), lung carcinoma cell line (A549), and human Caucasian breast adenocarcinoma (MCF7) (American Type Culture Collection). Cell viability is assessed by the mitochondrial-dependent reduction of yellow MTT (3-(4,5-dimethylthiazol-2-yl)-2,5-diphenyl tetrazolium bromide) to purple-blue insoluble formazan crystals [72].

#### 2.3.2. Procedure

All the following procedures were done in a sterile area using a Laminar flow cabinet biosafety class II level (Baker, SG403INT, Sanford, ME, USA). Cells were cultured in RPMI 1640 medium for HePG2, HCT116, and MCF7, while being cultured in DMEM for A549. The media were supplemented with 1% antibiotic-antimycotic mixture (10,000 U/cm^3^ Potassium Penicillin, 10,000 *μ*g/cm^3^ Streptomycin Sulfate, and 25 *μ*g/cm^3^ Amphotericin B), 1% L-glutamine, and 10% fetal bovine serum and kept at 37°C under 5% CO_2_ and 95% humidity.

Cells were batch-cultured for 10 days and then seeded at concentration of 1 × 10^4^ cells/well in fresh complete growth medium in 96-well microtiter plastic plates at 37°C for 24 h under 5% CO_2_ using a water jacketed carbon dioxide incubator (Sheldon, TC2323, Cornelius, OR, USA). Media were aspirated, fresh medium (without serum) was added, and cells were incubated either alone (negative control) or with different concentrations of sample to give a final concentration of (100, 50, 25, 12.5, 6.25, 3.125, 1.56, and 0.78 *μ*g/cm^3^). 0.5% DMSO and 100 *μ*g/cm^3^ of doxorubicin were used as negative and positive control, respectively. After 48 h of incubation, medium was aspirated, 40 mm^3^ MTT salt (2.5 *μ*g/cm^3^) were added to each well and incubated for further four hours at 37°C under 5% CO_2_. To stop the reaction and dissolving the formed crystals, a solution of 200 mm^3^ of 10% sodium dodecyl sulphate (SDS) in deionized water was added to each well and incubated overnight at 37°C. A positive control, doxorubicin that is composed of 100 *μ*g/cm^3^ was used as a known cytotoxic natural agent who gives 100% lethality under the same conditions [73].

The absorbance was then measured using a microplate multiwell reader (Bio-Rad Laboratories Inc., model 3350, Hercules, California, USA) at 595 nm and a reference wavelength of 620 nm. A statistical significance was tested between samples and negative control (cells with vehicle) using independent *t*-test by SPSS 11 program. DMSO is the vehicle used for dissolution of crystals of formazan and its final concentration on the cells was less than 0.2%. The percent cytotoxicity was calculated according to the formula:

(2) $$\begin{array}{c} \left[1-\left(\frac{\text{absorbance}\;\text{of}\;\text{treated}\;\text{cells}}{\text{absorbance}\;\text{of}\;\text{negative}\;\text{control}}\right)\right]\times 100. \end{array}$$

A probit analysis was carried for LC_50_ determination using SPSS 11 program.

## 3. Results and Discussion

### 3.1. FTIR Result

**Figure 1:**
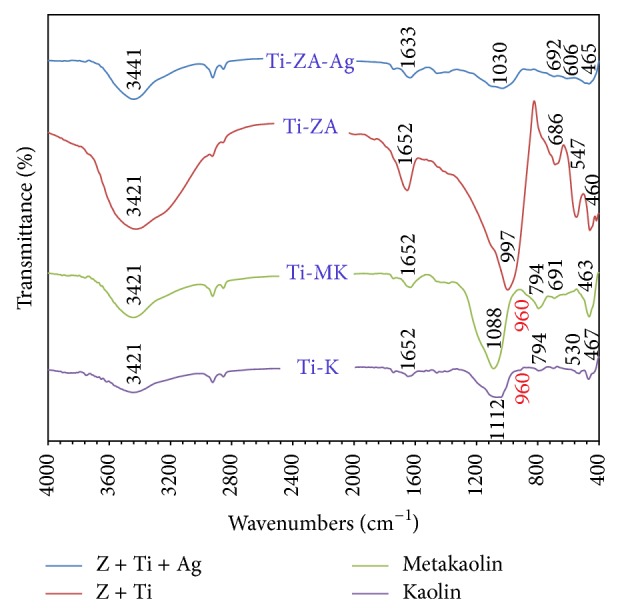
FTIR for the parent Kaolin before (Ti-K) and after (Ti-MK) calcination, Ti-Zeolite Na-A before (Ti-Z-A) and after silver substitution (Ti-Z-Ag).

The infrared spectrum of Kaolin coated with titanium tetrachloride is given in Figure 1. The mid-IR data show typical bands of Kaolin at 1112sh, 1091s, 1065s, and 1022s cm^−1^ representing the Si–O stretching vibrations, 794w cm^−1^ assign to *ν*_s_(Si–O–Si), 530m cm^−1^ represents Al^*VI*⁡^–O stretching vibration and, finally, 467m and 442sh cm^−1^ peaks are corresponding to the deformation vibration of Si–O [74].

Infrared bands (cm^−1^) for Ti-K, Ti-MK, Ti-Zeolite Na-A, and Ti-Zeolite Ag-A are given in Table 1.

On calcination, metakaolinite is formed with intense bands at 1088s and 794m cm^−1^, as the major feature. For metakaolinite, the disappearance of the 530m cm^−1^ band indicates the loss of Al[O(OH)]_6_ [75]. Bands which appear at 691w and 463sh may represent Anatase, which may have been formed during calcination.

Titanium-Zeolite prepared from titanium-metakaolinite is assigned by six main bands 997s {asymmetric stretching vibrations of bridge bonds *ν*_as_ Si–O(Si) and *ν*_as_ Si–O(Al)}, 686m {Anatase and symmetric stretching vibrations of bridge bonds *ν*_s_ Si–O–Si}, 547m {complex band, symmetric stretching vibrations of bridge bonds *ν*_s_ Si–O–Si and bending vibrations *δ* O–Si–O}, 460m {Anatase and bending vibrations *δ* O–Si–O occurring in antiphase} [76], 3421vb {O–H stretching}, and 1652m {O–H bending} cm^−1^  {associated with –OH absorption band, which is caused by physically adsorbed water or Zeolitic water} [77].

The FTIR spectra of titanium-Zeolite-A structure when treated for Ag-substitution show shifts in positions of wavenumbers: 997s to 1030b, 686m to 692m, 547m to 606m, and 460m to 465m cm^−1^, which improve the silver entry to the structural composition. Bands associated with water adsorbed by the Zeolite pores at 3421vb and 1652m cm^−1^ slightly shifted to 3441s and 1633m cm^−1^ due to the presence of van der Waals interactions between the hydroxyl groups in the Zeolite structure related to H_2_O and the positive charge on the surface of Ag^+^ [78]. The previous result improves the incorporation of silver ions into titanium-Zeolite structure.

The 960 cm^−1^ band is observed only in the IR-spectra of Al-containing Zeolites when the framework silica is substituted by titanium [79]. The previous band was firstly described as stretching vibration of a (SiO_4_) unit bonded to titanium atom or as a vibrational band of the Ti–O–Si fragment [80, 81]. However, after an extensive experimental IR and Raman studies, the band at about 960 cm^−1^ seemed to correspond to the stretching Si–O vibrational mode perturbed by the presence of titanium [79, 82].

In the present work, a small shoulder appears at about 960 cm^−1^ in the Kaolinite and metakaolinite substituted with titanium (Figure 1). The very thin layer of titanium coating the Kaolin particle may account for the weak signal of such band.

### 3.2. X-Ray Fluorescence

Table 2 shows the X-ray fluorescence analysis of both the starting Kaolinite and its Ti-treated form after calcination. The XRF data show clear increase in the Wt% of TiO_2_ from 2.26 in the parent Kaolinite to an amount of 7.18 in the coated metakaolinite, indicating the deposition of titanium on the precursor Kaolinite. In the same context, the ratio of SiO_2_/TiO_2_ suffers strong decrease from 22.36 in the uncoated to 7.42 in the coated samples. This probably suggests the incorporation of titanium in the Kaolinite structure.

We can also notice that the Wt% ratio of SiO_2_/Al_2_O_3_ shows slight increase from 1.60 in Kaolinite to 1.63 in the Ti-treated metakaolinite, which may account for the excess in quartz content of the treated calcined samples. Knowing that high acidic condition of the coating process turns Kaolinite less stable and titanium species highly reactive, this possibly facilitates the substitution of Si^+4^ by titanium cation in its position, with libration of an appreciable amount of silica which is then added to the quartz amount.

### 3.3. X-Ray Diffraction

**Figure 2:**
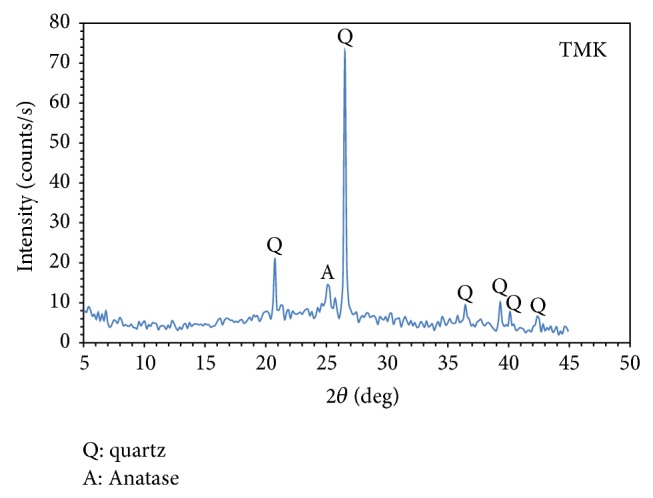
XRD pattern of the calcined Ti-containing Kaolin (Ti-metakaolinite) after calcination to metakaolinite (TMK) at 700°C/4 h.

Figure 2 represents the XRD pattern of calcined Ti-coated Kaolin when heated at 700°C to convert it to the activated metakaolinite (TMK), the amorphous precursor for Zeolite-Na-A. The obtained data shows very strong and sharp peaks of quartz (card# 05-0490), cocrystallizes with very small amount of crystalline TiO_2_ phase, Anatase (card# 71-1166). Anatase is a characteristic minor phase that normally developed as a secondary phase usually present in the parent kaolin. No other phases are present.

**Figure 3:**
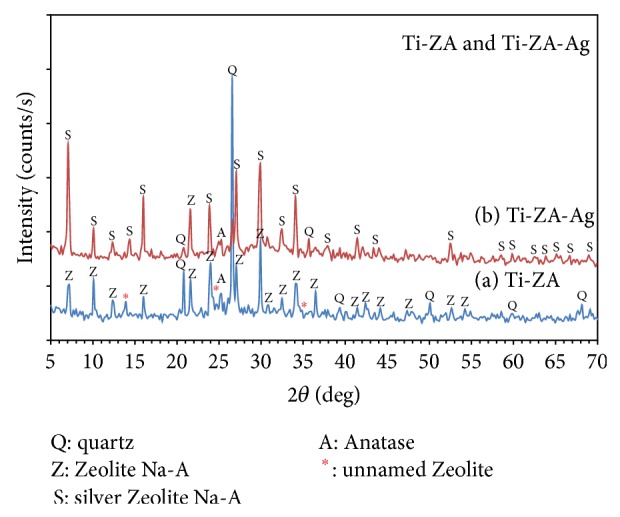
X-ray pattern for Ti-Zeolite (Ti-Z-A) and its silver-exchanged form (Ti-Z-Ag).

Figure 3 implies the XRD phases obtained in the synthetic product before (a) and after (b) Ag-substitution. Figure 3(a) represents the crystalline phases of Ti-Z-A, quartz, and Anatase which developed directly from the alkali attack of the Ti-metakaolinite at 80°C for 2 h. XRD pattern of all phases shows strong and sharp peaks with high intensities, indicating well crystallinity. There is a complete matching in the peak positions for both diffractograms of Zeolite-Na-A and its Ag-substituted form, which indicates the stability of Zeolite structure.

The semiquantitative analysis of the peak intensities of the obtained product (Figure 3(a)) shows a corresponding crystalline percent of 35% Zeolite-A (card# 39-0222), 41% quartz (card #05-0490), and 4.4% Anatase (card# 71-1166). Meanwhile, the XRD profile of the Ag-substituted product (Figure 3(b)) shows the cocrystallization of 37% Zeolite-A (card# 39-0222), 2.7% Ag-zeolite (card# 83-2089), 20.1% quartz (card # 05-0490), and 3.1% Anatase (card# 71-1166). In addition, the XRD screened the presence of an unidentified Zeolitic phase in the synthetic product with a characteristic main peaks appeared at* d*Å values of 6.35, 3.66, and 2.58 with some other minor peaks.

The previous results indicate the formation of an excess amount of free quartz in the synthetic product, which is much higher in its percent (44%) than that recorded in the starting Kaolin (20%). Furthermore, the crystallized Anatase (TiO_2_) is within the normal percentage of that usually formed in Zeolites developed from Kaolins. This may indicate the substitution of tetrahedral Si by Ti, a process that liberates silicon atoms responsible for the increase of quartz percentage.

The XRD data indicate the presence of 2.7% of Ag-zeolite in the Ag-substituted Ti-Z-A form, which supports the efficiency of the ion-exchanging process between Na^+^ and Ag^+^.

The semiquantitative analysis of the phases implies drastic decrease in the free quartz content from 44% in the Ti-Z-A (Figure 3(a)) to 20.1% in the Ti-Z-Ag sample (Figure 3(b)). The ion-exchanging process of Na^+^ by Ag^+^ releases sodium ions that may react with free quartz to form silicates [83], causing decrease in the free quartz content from 44% in Ti-Z-A to 20.1% in Ti-Z-Ag.

### 3.4. SEM and EDX

**Figure 4:**
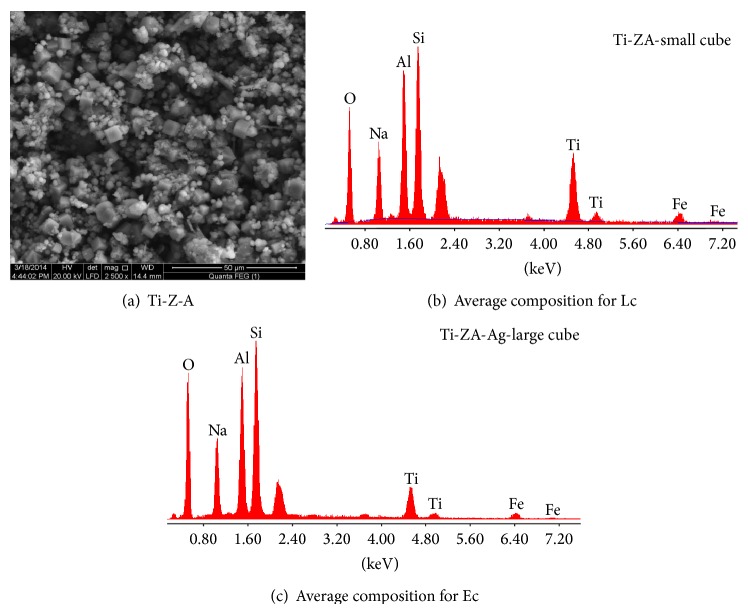
SEM micrographs for Ti-Z-A and its EDS analysis. Lc: late crystals = minute crystals. and Ec: early crystals = big crystals.

Figures 4(a), 4(b), and 4(c) represent the SEM micrographs for Ti-Zeolite Na-A (Ti-Z-A) and its EDS average surface chemical analysis (average of more than 5 different crystals of the nearly the same size). The micrographs show well-developed crystalline mixture consists of characteristic cubic-shaped crystals of Zeolite-Na-A type with sharp edges and average grain size of less than 5.0 *μ*m, copresent with large amount of minute rounded crystals of quartz. The microwave synthesis of oxides and powders usually yields products of relatively uniform crystals with narrow particle size distribution; however, in Figure 4(a), crystals seem related to different generations of development, having a wide range of grain size distribution expands from less than 0.25 to nearly about 5.0 *μ*m. This may be explained by the presence of different sizes of the metakaolinite particles in the reaction medium with different thickness of titanium coverage. In the reaction medium, thick titanium masking (shell) of the small metakaolinite core, relative to thinner shell over larger ones, may lead to delayed reaction between the metakaolinite core and the alkali solution, resulting in different rates of crystallization and/or generations.  Table 3 shows EDX analysis of “minute” and large cubes in Ti-Z-A.

Figures 4(b) and 4(c) and Tables 3 and 4 show the microanalysis for two extreme crystal generations: the early developed large crystals (Ec) and the lately formed minute crystals (Lc). Noticeably, both generations contain much higher Si/Al ratios (1.29 and 1.27 for Ec and Lc, resp.) than that recorded in the ideal Zeolite-Na-A type (1.03). Furthermore, the chemical composition of Zeolite product reveals clear reduction in their Si/Ti ratio (4.07 for Ec and 8.61 for Lc) from their reference Zeolite-Na-A (17.1), which means an increase in the Ti content of the formed Zeolite, especially for the very minute cubes. Minute cubes composition is regarded as good reflection of the chemical contents of Ti-metakaolinite precursor, thus, supporting the idea of substitution of silicon by titanium.

The Ag-exchanged Zeolite implies brighter crystals than those seen in Figure 4(a). The brighter appearance might be due to the interaction between the microscope beams of electrons and the incorporated silver within Zeolite structure.

**Figure 5:**
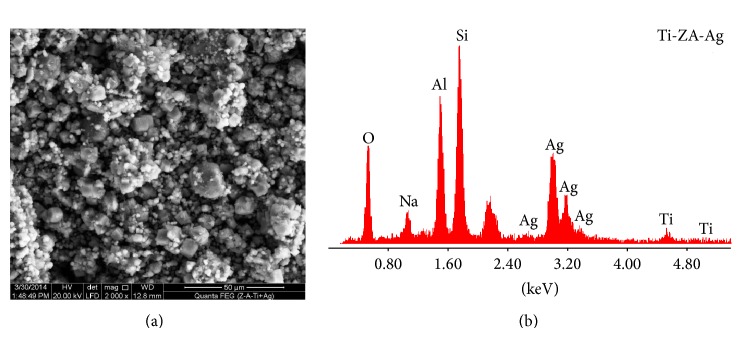
SEM and EDS chemical analysis for the Ag-substituted Ti-Z-A.

The SEM microstructure also shows that the Ti-Z-Ag crystals in Figure 5(a) show more rounded edges than Ti-Z-A crystals (Figure 4(a)).

Aluminosilicate Zeolite structures are unstable under acidic conditions and may suffer dealumination and even collapse [41]. This conclusion is supported by the XRD data, which showed reduction in the peak intensities of the crystalline Zeolite.

The Si/Al ratio also indicates an increase (1.42) than that recorded in the parent Kaolin and reference Zeolite, which again supports the idea of substituting Si by Ti in Zeolite structure. Titanium incorporated into the structure at the expense of silicon which is given off as free quartz increasing the Silicon content.

In the current study, the Wt% of silver (for initial AgNO_3_ concentrations of 0.1 M) exchanged onto the Zeolite determined by atomic absorption spectra (Savant AA, GBC, Australia) is 0.158% per Zeolite.

The EDS microanalysis of the Ti-Z-Ag given in Table 5 shows an obvious decrease in the At% content of Na^+^ from 13.5 in the Ti-Z-A into 4.72% in the Ag-exchanged form, indicating a partial substitution of Na^+^ by Ag^+^. The noticeable increase of Ag^+^ and detraction of Na^+^ ions confirms the efficiency of the ion-exchanging process between Na^+^ and Ag^+^, a result which the XRD result is confirming (Figure 3(b)).

In the present study, the concentration of the silver solution used in the exchanging process is in a diluted state of 0.1 M, which meets the minimum limit of AgNO_3_ concentration recommended for an efficient ion-exchange process [84]. The reason for maintaining the concentration of the silver solution at a diluted state is to prevent the oxidation of excess silver to silver oxide, which deposited in Zeolite pores that changes its effective porosity and surface area, affecting the availability of silver ions and reducing their activity.

### 3.5. *In Vitro* Cytotoxicity

*In vitro* cytotoxic activity evaluation of synthesized compounds (Ti-Z-Ag) was carried out against four human cancer cell lines including hepatocellular carcinoma (HePG2), colon carcinoma (HCT116), lung adenocarcinoma (A549), and breast adenocarcinoma (MCF7) using MTT method [72]. Doxorubicin HCl, which is one of the most effective antitumor agents, is used as a reference drug (positive control) in this study. The relationship between drug concentrations and cell viability is plotted to calculate LC_50_ (*μ*g/cm^3^) (lethal concentration of the sample that causes the death of 50% of cells in 48 h), the value which corresponds to the concentration required for 50% inhibition of cell viability.

**Figure 6:**
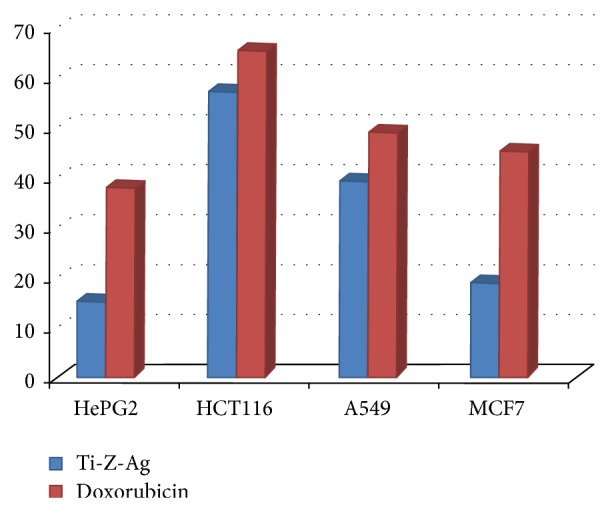
LC_50_ *μ*M of the prepared Ti-Z-Ag against carcinoma cell line compared with doxorubicin (positive control).

The screening summarized results in Table 6 and Figure 6 represent cytotoxic activity in terms of LC_50_ of Ti-Zeolite-A exchanged by Ag against the tested human cancer cell lines. Ti-Z-Ag causes death of 50% of tumor cells (LC_50_) at concentrations of 15.1 *μ*g/cm^3^ (37.8 for doxorubicin), 56.9 *μ*g/cm^3^ (65.1 for doxorubicin), 39.1 *μ*g/cm^3^ (48.8 for doxorubicin), and 18.8 *μ*g/cm^3^ (45.02 for doxorubicin) against HePG2, HCT116, A549, and MCF7, respectively. Cytotoxic activity of Ti-Z-Ag is tested against normal human epithelial amnion cells and the LC_50_ was 49 *μ*g/cm^3^, proving it is safe compound in comparison with doxorubicin.

The results revealed that cytotoxic activity of Ti-Z-Ag against HePG2, HCT116, A549, and MCF7 is astonishing and this safe compound can be considered after stages of clinical testing as a new antitumor drug.

Ag-NPs have the ability to inhibit angiogenesis, the pivotal step in tumor growth [85]. Compounds possessing antiangiogenic properties are known for their potential ability to block the activity of abnormally expressed signaling proteins, such as Ras and Akt, cytokine-based therapies, DNA- or protein-based vaccines against specific tumor markers, and tyrosine kinase inhibitors which exhibit a consistent antitumor effect [86].

The cytotoxic effects of silver are the result of active physical-chemical interaction of silver atoms with the functional groups of intracellular proteins, as well as with the nitrogen bases and phosphate groups in DNA [87]. It was also further implied that silver particles are directly toxic to the cancer cells through Caspase 3 activation [88], DNA fragmentation [89], and/or increased production of reactive oxygen species [90].

The actual pathways by which silver inhibits the pathway mediating cell proliferation and viability are yet to be explored.

## 4. Conclusion

In conclusion, novel method for the direct incorporation of Ti atoms into the structure of Kaolinite, the precursor of Zeolite-Na-A, is investigated in this study. The direct substitution of Si by Ti atoms in the tetrahedral position occurs. The scheme and mechanism are as follows.

The Kaolin sheets (Si and Al) are coated by Ti shell using titanium tetrachloride in a core-shell application process under acidic media. Under such acidic conditions, titanium cation is highly active, whereas Kaolinite structure is unstable. Thus, a proposed substitution of tetrahedral silicon by titanium can occur, resulting in Ti-Kaolinite. The calcination at high temperature of 700°C converts the Ti-Kaolinite to amorphous Ti-metakaolinite, the precursor of Ti-Zeolite Na-A. Finally, the alkali treatment of the Ti-precursor at 80°C/2 h under microwave hydrothermal conditions leads to Ti-Zeolite Na-A. Functionalization of Ti-Zeolite by silver results in Ag-Zeolite form. The cytotoxic activity of the Ag form of Ti-Zeolite Na-A is evaluated.

The evidences of Ti-Zeolite Na-A synthesis are summarized in the following points.

1. All testing tools, XRD, XRF, EDX, and IR measurements, indicate clear increase in the silicon content led to increase of Si/Al ratios in Ti-Kaolinite, metakaolinite, and prepared Zeolite. Desilication of the framework is proved to occur during tetanisation process of different Zeolite structures [40–43].
2. XRD for Kaolin, metakaolinite, and Zeolite witnessed clear increase in the free silica which accounts for the noticeable increase in the quartz content.
3. The increase of quartz content is an evidence of silica libration due its substitution by titanium.
4. In the coating process, the prevailing acidic conditions are turning Ti ions very active to replace the Si, resulting in the liberation of the substituted silica to be added to the free silica already present in the Kaolin. Thus, the XRD and EDX indicate stronger peaks of quartz as well as higher Si/Al ratio in the prepared Ti-Zeolite than unity.
5. The constant percent of Anatase (TiO_2_) phase in both the metakaolin and prepared Zeolite indicates a probable incorporation of Ti in the framework of Zeolite-A.
6. The obtained Si/Al and Si/Ti ratios for different generations of Zeolite crystals are higher than that of their parent Kaolin, supporting the idea of substituting Si by Ti. The concentration of silicon as free quartz increases as being replaced by titanium.

When compared with doxorubicin,* in vitro* cytotoxic result of Ag-substituted micronized Ti-Zeolite-A shows the highest efficiency against human hepatocellular carcinoma cell line (HePG2) followed by human Caucasian breast adenocarcinoma (MCF7) then lung carcinoma cell line (A549) and finally against colon cell line carcinoma (HCT116). Ti-Zeolite-A is a safe compound and can be considered a new antitumor drug.

## Figures and Tables

**Table 1:** Infrared bands (cm^−1^) for Ti-K, Ti-MK, Ti-Zeolite Na-A, and Ti-Zeolite Ag-A.

Kaolin	Metakaolin coated with titanium	Zeolite incorporated with titanium	Zeolite incorporated with titanium and silver
1112sh, Si–O	1088s, Si–O	997s, Si–O(Si), and Si–O(Al)	1030b, Si–O(Si), and Si–O(Al)
1091s, Si–O	794m, Al–O	686m, Si–O–Si, and Anatase	692m, Si–O–Si, and Anatase
1065s, Si–O	691w, Anatase	547m, Si–O–Si, and O–Si–O	606m, Si–O–Si, and O–Si–O
1022s, Si–O	463sh, Anatase	460m, O–Si–O, and Anatase	465m, O–Si–O, and Anatase
794w, Si–O–Si	794sh, Si–O–Si	3421vb, O–H	3441s, O–H
530m, Al^VI^–O	530sh, Al^VI^–O	1652m, O–H	1633m, O–H
467m, Si–O			
442sh, Si–O			

**Table 2:** Chemical analysis of the starting Kaolinite and Ti-treated form [37].

Oxides	Kaolinite Wt%	Ti-coated metakaolinite
SiO_2_	50.54	53.33
Al_2_O_3_	31.48	32.64
TiO_2_	2.26	7.18
Fe_2_O_3_	1.87	1.97
MgO	0.90	1.02
ZnO	0.01	0.06
CaO	0.66	0.85
Na_2_O	0.10	0.11
K_2_O	0.07	0.07
P_2_O_5_	0.08	0.09
SO_3_	0.08	0.02
Cl	0.02	0.23
L.O.I	11.75	2.40

Total	100	100

**Table 3:** EDX analysis for Ti-Zeolite (Ti-ZA).

Element	Minute cubes		Large cubes	
	Wt%	At%	Wt%	At%
O K	36.14	50.38	40.50	53.95
NaK	13.03	12.63	13.50	12.52
Al K	17.29	14.29	17.82	13.65
Si K	22.03	17.49	22.92	17.39
Ti-K	9.23	4.30	4.55	2.02
Fe K	2.27	0.91	1.25	0.48

Total	100.00	100	100	100

**Table 4:** EDX percentage of atomic constituents (At%) for starting precursor (Ti-MK), reference Zeolite, and Ti-Zeolite crystals of different generations.

At.% ratio	Ti-MK^*^	Ref. Zeolite^**^	Ti-Zeolite	
			Minute cube	Large cube
Si/Al	1.28	1.03	1.29	1.27
Si/Ti	5.72	17.11	4.07	8.61

^*^Ti-metakaolinite, that is, Ti-coated Kaolin after calcination (EDS analysis is not included).

^**^Zeolite prepared from the starting kaolin before coating with titanium.

**Table 5:** EDX microanalysis of the Ag-substituted Ti-Z-A.

Element	Wt%	At%
O K	28.20	50.03
NaK	3.83	4.72
Al K	14.07	14.81
Si K	20.82	21.04
Ti-K	2.08	1.23
Ag K	31.01	8.16

Total	100	100

**Table 6:** Cytotoxic screening of the novel synthesized Ti-Zeolite-A exchanged by Ag against the tested human cancer cell lines.

Human cancer cell lines	Ti-Zeolite-Ag	^*^Doxorubicin
	LC_50_ (*µ*g/cm^3^)	LC_50_ (*µ*g/cm^3^)
HePG2	15.1	37.8
HCT116	56.9	65.1
A549	39.1	48.8
MCF7	18.8	45.02

^*^Positive control adriamycin (doxorubicin).
